# Retrieval of refractivity fields from GNSS tropospheric delays: theoretical and data-based evaluation of collocation methods and comparisons with GNSS tomography

**DOI:** 10.1007/s00190-024-01903-9

**Published:** 2024-11-30

**Authors:** Endrit Shehaj, Alain Geiger, Markus Rothacher, Gregor Moeller

**Affiliations:** 1 Institute of Geodesy and Photogrammetry, ETH Zürich, Zurich, Switzerland; 2 TU Wien, Department of Geodesy and Geoinformation, Vienna, Austria

**Keywords:** GNSS meteorology, Troposphere, Collocation adjustment, GNSS tomography

## Abstract

This paper focuses on the retrieval of refractivity fields from GNSS measurements by means of least-squares collocation. Collocation adjustment estimates parameters that relate delays and refractivity without relying on a grid. It contains functional and stochastic models that define the characteristics of the retrieved refractivity fields. This work aims at emphasizing the capabilities and limitations of the collocation method in modeling refractivity and to present it as a valuable alternative to GNSS tomography. Initially, we analyze the stochastic models in collocation and compare the theoretical errors of collocation with those of tomography. We emphasize the low variability of collocation formal variances/covariances compared to tomography and its lower dependence on a-priori fields. Then, based on real and simulated data, we investigate the importance of station resolution and station heights for collocation. Increasing the network resolution, for example, from 10 to 2 km, results in improved a-posteriori statistics, including a 10% reduction in the error statistic for the retrieved refractivity up to 6 km. In addition, using additional stations at higher altitudes has an impact on the retrieved refractivity fields of about 1 ppm in terms of standard deviation up to 6 km, and a bias reduction of more than 3 ppm up to 3 km. Furthermore, we compare refractivity fields retrieved through tomography and collocation, where data of the COSMO weather model are utilized in a closed-loop validation mode to simulate tropospheric delays and validate the retrieved profiles. While tomography estimates are less biased, collocation captures relative changes in refractivity more effectively among the voxels within one height level. Finally, we apply tomography and collocation to test their capabilities to detect an approaching weather front. Both methods can sense the weather front, but their atmospheric structures appear more similar when the GNSS network has a well-distributed height coverage.

## Introduction

Modeling atmospheric water vapor poses a considerable challenge due to its spatio-temporal variations. Hence, there is a pressing need to explore novel sensing techniques for three-dimensional mapping of water vapor distribution to advance our understanding of the current atmospheric state and its temporal evolution within the Earth's troposphere. Microwave satellite signals, as those of GNSS (Global Navigation Satellite Systems), are sensitive to the constituents along the transmitter/receiver path. Depending on the atmospheric conditions along the signal path, they experience different delays and distortions. Considered as a nuisance in geodetic applications (such as navigation), these delays can provide valuable information regarding the distribution and amount of gases in the atmosphere. While the dry gases exhibit slow variations in both, space and time, allowing for relatively accurate modeling and prediction of their distribution (and subsequent delay on microwave signals), the modeling of wet refractivities introduces larger uncertainties. Consequently, for highest accuracy, the wet delay is estimated during the processing of microwave measurements from geodetic techniques. This information provides knowledge regarding the distribution of water vapor. It can be used for the correction of observations carried out by space geodetic techniques, as well as for meteorological applications (Geiger 1987; Bevis et al. 1992; Yuan et al. 1993; Davis et al. 1996; Elgered et al. 1997; Kruse 2001; Troller 2004; Hurter 2014). The assimilation of GNSS tropospheric products into numerical weather prediction (NWP) models and their benefits have been reported in many research publications, such as (Poli et al. 2007; Bennitt and Jupp 2012; Lindskog et al. 2017; Nakamura et al. 2004).

The most basic tropospheric parameter that ground-based GNSS provides is the zenith total delay (ZTD), estimated nowadays with sub-cm level accuracy (Teunissen and Montenbruck 2017). Further advancements in GNSS processing and modeling have led to the estimation of ZTD gradients, which represent the north–south and east–west tilt, i.e., the azimuthal asymmetry of the atmosphere (Ning and Elgered 2021). Another non-trivial parameter is the residual tropospheric delay. This residual of the least-squares adjustment is assumed to hold part of the unmodeled neutral atmosphere and, thus, contains information regarding local anisotropic tropospheric effects. However, this information is poorly understood (Hurter 2014; Jones et al. 2020). An improved parameter derived from these estimates is the slant total delay (STD). It represents the delay in the direction from the receiver to the transmitter (Moeller and Weber 2015). STDs are computed by mapping the ZTDs into the slant direction, also incorporating zenith gradients and possible residuals retrieved during the processing of GNSS phase observations (Moeller and Weber 2015; Alshawaf 2013).

In the past decades, GNSS tomography has become more and more attractive, motivated by the densification of GNSS networks, improved modeling strategies and high temporal resolution of GNSS observations. Modeled refractivities are quantities with a better interpretability for the meteorological community and can depict 3D tropospheric structures. Several studies have been conducted, and GNSS tomography software packages have been developed and maintained for over a decade now (Kruse 2001; Troller 2004; Champollion et al. 2005; Perler 2012; Heublein 2019; Möller 2017). The main idea behind GNSS tomography lies in the simple fact that the slant delay is the integral of the refractivity along the path from the GNSS satellite to the receiver's antenna; above 15 degrees elevation, the ray bending can be ignored (Ichikawa et al. 1995; Möller and Landskron 2019). The atmosphere around the GNSS network is discretized in a 3D grid of voxels, where the refractivity N can be assumed as constant or modeled to change according to a simple model, namely, node-based parametrization; this helps to avoid sudden jumps of refractivity values between neighboring voxels. A simple tomography solution would be possible in a scenario where non-colinear rays cross the cells and where at least one ray is crossing each cell, and even better if several rays would cross each voxel. Unfortunately, due to the limited number of slant observables, this is usually not the case. A regularization method should be applied to the inversion process to ensure a stable and unique solution (Möller 2017; Adavi 2022).

Although GNSS tomography has limitations related to station density and number of observations, different works have proven its capability to retrieve appropriate refractivity fields. (Bender et al. 2011) show that the tomography solution can be further improved by adding additional constraints, such as integrating estimates of integrated water vapor (IWV). (Rohm et al. 2014) use a Kalman filter scheme and remove linearly dependent observations and parameters, achieving an accuracy of 4.2 and 6.2 ppm for simulated and real data, respectively, compared to NWP refractivity. (Brenot et al. 2017) focus on numerical weather forecasts to initialize GNSS tomography, showing the possibility of improved nowcasting scenarios. At ETH-IGP, several campaigns have been carried out to compare tomography with radiosonde-retrieved refractivities; they showed that accuracies of few ppm are reachable by means of tomography (Kruse 2001; Troller 2004; Hurter 2014; Perler 2012). In parallel, a least-squares collocation software COMEDIE (Collocation of Meteorological Data for Interpretation and Estimation of Tropospheric Path Delays) has been developed and deployed since the beginning of the 90s (Troller 2004; Eckert et al. 1992a, 1992b; Geiger and Cocard 1992; Hirter 1998). COMEDIE can successfully interpolate and extrapolate meteorological parameters such as temperature, pressure, water vapor pressure, tropospheric zenith delays and delay gradients as well as refractivities in four dimensions (Troller 2004; Hurter 2014; Eckert et al. 1992a, 1992b; Hirter 1998; Wilgan et al. 2017). More recently, it has been further upgraded to collocate (relative) slant delays, such as InSAR unwrapped phase differences (Shehaj et al. 2020).

COMEDIE models the path delays as the sum of a functional part, a stochastic part named 'signal,' and a 'noise' part which is Gaussian white noise. The functional part is based on simple physical principles, and the ‘signal’ is determined empirically and considers the distances between the observations. Hence, the 'signal' and the 'noise' terms represent correlated and uncorrelated noise in the residuals, respectively. The refractivity is described using the same parameters as for the zenith delays by deriving the formulas of the functional and stochastic parts accordingly. This is an alternative approach to tomography, which produces fields of refractivity based on estimation and stochastic modeling. An advantage of the collocation method compared to tomography is that the limited number of measurements does not hamper the solution. However, more measurements lead to more accurately estimated parameters, and the empirically determined stochastic part is more realistic. The accuracy of collocation is clearly dependent on the accuracy of the deterministic and stochastic models, which have been validated through several works over the past decades.

In this work, we focus on retrieving refractivity fields from collocation, which we consider an alternative to tomography. Our main objective is to emphasize the retrieval of tomography-like products using collocation to the GNSS meteorology community, by displaying the benefits and limitations of this technique.

For this purpose, we present a theoretical evaluation of our models. We also assess the impact of network density and network height range on our results. In addition, we provide a theoretical comparison between tomography and collocation in terms of formal variances/covariances. Thereby we apply collocation to two real datasets, collected at locations with very different topographies, and compare the retrieved fields as well as investigate the observation residuals. Furthermore, we present a closed-loop validation based on NWP model data, where we evaluate refractivity fields produced by tomography and collocation. Finally, we apply collocation and tomography to a scenario, where a weather front crosses the GNSS network, with the purpose to assess their capabilities of detecting the atmospheric structures under such conditions.

This work aims to show that using collocation to retrieve refractivity fields from GNSS delays leads to results comparable to GNSS tomography. The two methods are compared theoretically and experimentally; this is a new aspect addressed here. We also provide a comprehensive analysis of different aspects that affect the results of collocation such as the evaluation of the stochastic models and the quantification of the impact of network density and distribution of the heights in the GNSS network.

The paper is organized as follows. Section 2 provides a summary of the theoretical background of collocation and tomography and describes the datasets used in this work. Section 3 provides a theoretical and data-based comparison of collocation and tomography and investigates the importance of various factors that might affect the collocation results, such as fine-tuning of the models and the distribution of GNSS stations in the network. Section 4 summarizes the major findings and discusses further points that have emerged from this work.

## Methods and datasets

This section provides a description of the main methods employed in this study for retrieving refractivity fields: GNSS tomography and collocation. In addition, we provide the mathematical formulations of the equations used to investigate the formal variances/covariances and the stochastic model of collocation. The section concludes with a description of the datasets used in our analysis.

### Mathematical background: GNSS tomography

GNSS tomography is a technique that reconstructs the water vapor distribution in space and times, using slant and zenith delays. Thereby water vapor structures at different heights are resolved. Tomography approaches have been developed based on least squares (for instance (Möller 2017)), Kalman filter (for instance (Perler 2012)) or algebraic reconstruction technique (for instance (Zhang et al. 2020)).

The main principle in tomography is, that the path delay ($PD$) can be expressed as a function of the refractivity in the tomographic voxels it crosses, and the geometric length it travels in each voxel:

1 $$ PD_{j} = 10^{ - 6} \cdot \mathop \sum \limits_{i = 1}^{k} N_{i} \Delta s_{i,j} $$

where $\Delta {s}_{i,j}$ is the length of the ray *j* in the voxel *i*, and $k$ is the total number of voxels crossed by the ray. The tomography equation consisting of all rays and refractivities, is defined as follows:

2 $$ PD = A \cdot N $$

where $PD$ represents the vector of all path delay observations ${PD}_{j}$, $N$ is the vector of refractivities for all voxels and the matrix $A$ contains the partial derivatives of the slant delays with respect to the refractivities, resulting in the ray lengths $\Delta {s}_{i,j}$ in each voxel.

The main challenge in GNSS tomography is to form a regular normal equation matrix that can be inverted. This is a necessary condition if the quantity of interest in each voxel should be determined from the rays crossing it. It is restricted by the fact that measurements can only be acquired within a limited observation window defined by the satellite–receiver geometry. With the current infrastructure, it is usually not possible to have enough satellites and ground stations to allocate observations to each voxel. As a consequence, GNSS tomography is an example of an ill-posed problem, where many voxels are underdetermined or overdetermined. According to Menke (1989), the whole system is mixed-determined, and this may lead to singularities. Singular value decomposition is a typical approach to deal with this issue, where underdetermined and overdetermined voxels are separated. In addition, voxel constraints are an alternative to resolve this issue, where an a-priori field is assumed or the specific behavior of neighboring voxels is modeled. The weight of the a-priori background field must be treated and constrained properly in the system, since the final solution depends on it. (Adavi 2022) has shown that the direct regularization method (TV) can be used for a successful solution without requiring an a-priori field. The ill-posed problem of GNSS tomography is further pronounced by the often ill-conditioned system equations leading to instable solutions. Slight changes in the measurements can cause extremely unstable parameter solutions. To ensure a stable and unique solution, a regularization method, such as singular value decomposition (SVD), should be applied to the inversion process (Möller 2017).

The SVD method allows for resolving singular and non-square matrices (Strang and Borre 1997). Therefore, the design matrix $A$ is split into three components as follows:

3 $$ A = U \cdot S \cdot V^{T} $$

where $U\left(1:l,1:l\right),$ with $l$ the number of observations, and ${V}^{T}(1:m,1:m)$, with $m$ the number of voxels, are orthogonal matrices. $U$ and ${V}^{T}$ are the normalized left and right singular vectors of $A$. $S(1:l,1:l)$ is a diagonal matrix where the diagonal elements are the singular values, while $S(1:l,l+1:m)$ has zero elements.

First, we find the rank $r$ of the observation matrix $A$, and then, we can define the resolution matrix $R$ as follows:

4 $$ R = {\text{diag}}\left( {V_{r} \cdot V_{r}^{T} } \right) $$

where ${V}_{r}$ is equal to $V(1:m,1:r)$. The resolution matrix is used to define which voxels can be resolved. (Adavi et al. 2022) propose to use the spread of the resolution matrix to analyze the results of the retrieved field. It shows correlation between the spread and the error of the retrieved field in terms of standard deviation. They conclude that this parameter could be used as a quality indicator to pre-assess the performance of the retrieved field or to be used a-posteriori to validate the solution.

When we introduce an a-priori field ${N}_{apr}$, the system matrix and the observations matrix change as follows:

5 $$ A = \left[ {A; A_{apr} } \right] $$

6 $$ OBS = \left[ {PD; N_{apr} } \right] $$

where ${A}_{apr}$ is an identity matrix, and the standard deviation of the a-priori field is considered in the weight matrix.

According to Eq. (1), the field is assumed constant within a voxel, which is the simplest parametrization. (Perler 2012) introduced trilinear and a combined bilinear/spline parameterization to improve the quality of the retrieved refractivity fields. Further advancements in parameterization have been studied in Adavi (2022), where Eikonal Ray-Tracing methods were compared to straight line geometries. (Ding et al. 2018) introduce an adaptive node parameterization approach, for varying density on different tomographic planes. In addition, to discretize the tomographic field for each plane at each epoch, the size of the tomographic field is used to determine the location and number of nodes on the plane. (Trzcina et al. 2023) propose a new parameterization approach, where they account for the non-uniform distribution of the GNSS observations to optimize the locations of the tomographic nodes.

In this work, we use the GNSS tomography methods described in this section to evaluate the formal variances/covariances of tomography, where the resolution matrix is used as criteria to define if a voxel can be resolved; the results are displayed in Sect. 3.2. The refractivity fields presented in Sect. 3.1 are retrieved using the tomography software ATom, which is based on least-squares estimation. For further details about the ATom software, we refer to Möller (2017).

### Mathematical background: least-squares collocation of ZTDs and refractivities

Least-squares collocation is an enhancement to the traditional adjustment theory. Apart from the estimation of some parameters describing the deterministic part, the residuals are exploited to estimate a so-called signal, which represents correlated noise. The remaining part of the residuals is considered white uncorrelated noise. Each measurement $l$ is thus described as follows (for example, from Troller (2004)):

7 $$ l = f\left( {u,x,t} \right) + s\left( {C_{ss} ,x,t} \right) + \in $$

with $f$, $s$ and $\epsilon$ representing the functional, signal and noise part, respectively. $u$ represents the vector of parameters to be estimated, and $x$ and $t$ are the coordinates and time, respectively. The signal is also described by the covariance ${C}_{ss}$ designed empirically to describe the correlations among the measurements.

The functional part of zenith total delays is described as follows:

8 $$ ZTD\left( {u,x,y,h,t} \right) = \left[ {ZTD_{0} + a_{ZTD} \cdot \left( {x - x_{0} } \right) + b_{ZTD} \cdot \left( {y - y_{0} } \right) + c_{ZTD} \cdot \left( {t - t_{0} } \right)} \right] \cdot e^{{ - \frac{{h - h_{0} }}{{H_{ZTD} }}}} $$

where $u=\left({ZTD}_{0},{a}_{ZTD},{b}_{ZTD},{c}_{ZTD},{H}_{ZTD}\right)$ represents the vector of parameters to be estimated. $x$ and $y$ are the east and north coordinates, $h$ is the height and $t$ is the time of a measurement. The parameters ${x}_{0}$,${y}_{0}$,${h}_{0}$ and ${t}_{0}$ are the coordinates and time of an arbitrary chosen reference point inside the network (usually the mean of north, east, time coordinates and height ${h}_{0}$ is set to 0). Therefore, ${ZTD}_{0}$ is the delay at the reference position, ${H}_{ZTD}$ is the atmospheric scale height (distance over which the *ZTD* decreases by a factor of the Euler number $e$) and ${a}_{ZTD}$, ${b}_{ZTD}$ and ${c}_{ZTD}$ are the gradients in $x, y$ and $t$, respectively. In case of a bad distribution of GNSS measurements, for example, in height (such as when one station of the network is at located above 3 km and all the other stations are located below 1 km), COMEDIE discards the observations that would not fit well the model based on gross-error detection (Eckert et al. 1992a).

The covariance matrix describing the signal is modeled as follows:

9 $$ C_{ss} \left( {i,j} \right) = \frac{{\sigma_{0}^{2} }}{{1 + \left[ {\left( {\frac{{x_{i} - x_{j} }}{{\Delta x_{0} }}} \right)^{2} + \left( {\frac{{y_{i} - y_{j} }}{{\Delta y_{0} }}} \right)^{2} + \left( {\frac{{h_{i} - h_{j} }}{{\Delta h_{0} }}} \right)^{2} + \left( {\frac{{t_{i} - t_{j} }}{{\Delta t_{0} }}} \right)^{2} } \right] \cdot e^{{ - \frac{{h_{i} + h_{j} }}{{2h_{0} }}}} }} $$

with $\left({x}_{i},{y}_{i},{h}_{i},{t}_{i}\right)$ and $\left({x}_{j},{y}_{j},{h}_{j},{t}_{j}\right)$ representing the coordinates and times of two measurements. $\Delta {x}_{0}$, $\Delta {y}_{0},\Delta {h}_{0}$ and $\Delta {t}_{0}$ are the signal correlation lengths in space and time, while ${\sigma }_{0}$ and ${h}_{0}$ are the a-priori covariances of the signal and the scale height modifying the signal correlation lengths as a function of height. In (Hurter 2014), it is explained how the parameters of the covariance matrix can be determined from the data.

The deterministic part of the refractivity is computed by taking the derivative of the *ZTD* model with respect to the zenith direction (Hurter 2014):

10 $$\begin{aligned}& N\left( {u,x,y,h,t} \right) = \frac{1}{{H_{ZTD} }}\cdot\\ &\left[ {ZTD_{0} + a_{ZTD} \cdot \left( {x - x_{0} } \right) + b_{ZTD} \cdot \left( {y - y_{0} } \right) + c_{ZTD} \cdot \left( {t - t_{0} } \right)} \right] \\ & \qquad \cdot e^{{ - \frac{{h - h_{0} }}{{H_{ZTD} }}}} \end{aligned}$$

The formulation of the covariance of the signal, describing the relation between the measurement ($$ZTD)$$ and the interpolated refractivity ($$N)$$ is shown in Hurter (2014); Wilgan et al. 2017, once more obtained by taking the derivative of the model for ZTDs with respect to the zenith direction. Therefore, after the collocation of the delays, the refractivity can be modeled at any point of the area of investigation, using the same functional and signal parameters as for the delays, but with different (and slightly more complex) formulations.

### Formal variances/covariances analysis

In this section, we provide the mathematical formulation of the formal variances/covariances of tomography and collocation. To compute these errors, the observation (design) matrix and the variance/covariance matrices of the measurements are utilized. The formal variances/covariances show the expected inaccuracies in the resolved fields due to the geometry (network configuration) and the a-priori knowledge on the accuracy of the GNSS observations. In this work, we have defined a 10 × 10 grid and simulated different network geometries to evaluate/compare the errors for both methods. Therefore, we do not need to simulate the measurements themselves, but only their ray paths within the grid. The respective results are shown in Sect. 3.2.

The formal variances/covariances of the tomography solution (for the retrieved refractivity) are obtained as follows:

11 $$ E_{xx} = \left( {A^{T} D^{ - 1} A} \right)^{ - 1} $$

where A is the tomography design matrix, and $D$ is the covariance matrix of the measurements.

The formal variances/covariances of the collocation approach (for the retrieved refractivity) are provided by the following equation (Wirth 1990):

12 $$ E_{tt} = C_{s^{\prime}s^{\prime}} - C_{s^{\prime}s} D^{ - 1} C_{s^{\prime}s}^{T} + \left( {HA - A_{1} } \right)E_{xx} \left( {A^{T} H^{T} - A_{1}^{T} } \right) $$

where $D= {C}_{nn}+{C}_{ss}$ is the sum of the covariance of the signal and the covariance of the measurements, and $H= {C}_{s{\prime}s}{D}^{-1}$. ${C}_{s{\prime}s{\prime}}$ is the signals' covariances between the values of $N$ in the retrieved refractivity fields, ${C}_{s{\prime}s}$ is the covariance between the refractivity values $N$ and the $ZTD$ measurements and ${C}_{ss}$ is the covariance between the $ZTD$ measurements. $A$ and ${A}_{1}$ are the design matrices of the collocated measurements ($ZTD$) and the retrieved refractivities ($N$), respectively.

Based on Eqs. 11 and 12, we perform a 2D evaluation of the a-priori errors of tomography and collocation. For tomography, we ray trace the signals within our 2D (east and height) voxel model. For collocation, we consider the following simplified functional relations for the zenith total delays (Eq. 13) and the refractivities (Eq. 14), respectively:

13 $$ ZTD\left( {x,y,h,t} \right) = [ZTD_{0} + a_{ZTD} \left( {x - x_{0} } \right)]e^{{ - \frac{h}{{H_{ZTD} }}}} $$

14 $$ N\left( {x,y,h,t} \right) = \frac{1}{{H_{ZTD} }}\left[ {ZTD_{0} + a_{ZTD} \left( {x - x_{0} } \right)} \right]e^{{ - \frac{h}{{H_{ZTD} }}}} $$

For the stochastic modeling, the following equations are considered:

15 $$ C_{ss} \left( {ZTD_{i} ,ZTD_{j} } \right) = \frac{{\sigma_{0}^{2} }}{{1 + \left[ {\left( {\frac{{x_{i} - x_{j} }}{{{\Delta }x_{0} }}} \right)^{2} + \left( {\frac{{h_{i} - h_{j} }}{{{\Delta }h_{0} }}} \right)^{2} } \right]}} $$

16 $$ C_{ss} \left( {N,ZTD} \right) = \frac{{\sigma_{0}^{2} }}{{q^{2} }}\left[ {\frac{{2\left( {h_{N} - h_{ZTD} } \right)}}{{\left( {\Delta h_{0} } \right)^{2} }}} \right] $$

17 $$ C_{ss} \left( {N_{i} ,N_{j} } \right) = \frac{{2\sigma_{0}^{2} }}{{q^{2} }}\left[ {\frac{1}{{\left( {\Delta h_{0} } \right)^{2} }} - \frac{{4\left( {h_{i} - h_{j} } \right)^{2} }}{{q\left( {\Delta h_{0} } \right)^{4} }}} \right] $$

18 $$ q = 1 + \left[ {\left( {\frac{{x_{i} - x_{j} }}{{\Delta x_{0} }}} \right)^{2} + \left( {\frac{{h_{i} - h_{j} }}{{\Delta h_{0} }}} \right)^{2} } \right] $$

### Fine-tuning of the stochastic collocation model for refractivity estimation

This section provides the mathematical formulations used to evaluate the stochastic model in collocation. We discuss the models separately for the height and the east/north/time components. The results of the fine-tuning are presented and further analyzed in Sect. 3.3.1.

The innovation of collocation compared to classical least squares is the stochastic model, which takes a correlated noise into account. Our stochastic models are defined empirically and have been studied and validated over the course of several works (Kruse 2001; Troller 2004; Hurter 2014; Hirter 1998; Wilgan et al. 2017; Shehaj et al. 2020). Apart from the validation of the models with several test campaigns, in this paragraph, we evaluate our models in a more analytical approach, by utilizing NWP data.

#### Stochastic model in the height component

The decrease of the ZTD with increasing height is well described by an exponential function. Therefore, to evaluate our height assumption, we derive our stochastic functions from the following simplified model:

19 $$ N\left( h \right) = \frac{1}{{H_{ZTD} }} \cdot ZTD_{0} \cdot e^{{ - \frac{h}{{H_{ZTD} }}}} $$

Equation (19) shows that the functional part of refractivity decreases exponentially. In this evaluation, we are mainly interested in how well our empirical stochastic functions can model correlated noise in the height component, after removing the functional part. For this purpose, we utilize an NWP model and fit an exponential function, which we consider to be modeled by the functional part. Therefore, we obtain residuals of the NWP profiles, which we use to evaluate our stochastic functions for covariance (Eq. (20)) and correlation (Eq. (21)), respectively:

20 $$ Cov\left( {N_{{h_{1} }} ,N_{{h_{2} }} } \right) = \frac{{2l_{h}^{2} \left( {l_{h}^{2} - 3r^{2} } \right)}}{{\left( {l_{h}^{2} + r^{2} } \right)^{3} }} $$

21 $$ Corr_{{N\left( {h_{1} } \right),N\left( {h_{2} } \right)}}^{r} = \frac{{l_{h}^{4} \left( {l_{h}^{2} - 3r^{2} } \right)}}{{\left( {l_{h}^{2} + r^{2} } \right)^{3} }} $$

with $l_{h}$ as the correlation length in height and $r = \left| {h_{1} - h_{2} } \right|$ as the height difference between two points.

Section 3.3 further analyzes the correlations between the stochastic model and the NWM residuals (after fitting an exponential function) in the height component.

#### Stochastic model in the north/east/time component

As we can see from Eq. (10), our functional model is similar for the north, east and time coordinates. Therefore, we only consider the east component and the following simplification, to evaluate our model in the east component.

22 $$ N\left( x \right) = \frac{1}{{H_{ZTD} }}\left[ {ZTD_{0} + a_{ZTD} \cdot x} \right] $$

The simplified signal correlation formulation is the same as for the height, with the correlation length $l_{x}$ and $r = \vert x_{1} - x_{2} \vert$:

23 $$ Corr_{{N\left( {x_{1} } \right),N\left( {x_{2} } \right)}}^{r} = \frac{{\left. {l_{x}^{4} (l_{x}^{2} - 3r^{2} } \right)}}{{\left( {l_{x}^{2} + r^{2} } \right)^{3} }} $$

Similarly to the previous section, we remove a linear component from the NWP refractivities in the east component. Therefore, we can evaluate the fit of the stochastic model in the east component compared to the residuals of the NWP data. Here, we use a fixed height of 4.5 km since the data we use stems from a mountainous area, in the Valais, and some profiles start at altitudes of ~ 4 km. Since the NWP model layers do not have iso-heights, we interpolate all the profiles at 4.5 km. Thus, we use a simple interpolation function, as the NWP model is quite dense in the height component (see Sect. 2.5.1).

### Datasets and test area

#### NWP data

We used the NWP model COSMO-1 (Consortium for Small-scale Modeling) to simulate zenith and slant delays. It is operated by MeteoSwiss to produce weather forecast in the Alpine region (MeteoSwiss 2019). In this work, we used a grid of 65 vertical layers up to ~ 11 km, where pressure, water vapor pressure and temperature are provided hourly. The horizontal resolution is 1.1 km. We ray-traced through the NWP model and used COMEDIE to interpolate the atmospheric variables (pressure, temperature and dry pressure) with a resolution of 300 m along the slant and zenith paths. The interpolation of meteorological parameters with COMEDIE is described in Troller (2004); Eckert et al. 1992a; Eckert et al. 1992b. Since the COSMO grid has a very high resolution, the error due to interpolation with COMEDIE is very small. Therefore, we calculated the refractivity and finally integrated it to calculate the delays. The delay above the COSMO grid is modeled using the Saastamoinen model (Saastamoinen 1973).

For our analysis, we simulated NWP model-based slant and zenith delays for the Valais network. The elevation and azimuth of GPS and GLONASS satellites in view were derived from broadcast ephemerides using the software ATom (Möller 2017). The slant delays entering the NWP model through any lateral surface were not simulated. In addition, we also simulated very high-resolution GNSS networks based on the grid of the COSMO model. It allows for testing of different resolutions of stations that are equally distributed in space, and, therefore, verify the effect of the network resolution in a closed-loop validation. In subsequent sections, the refractivity of the COSMO model is used as ground truth to depict the collocation and tomography a-posteriori errors.

Similarly, based on the COSMO grid, we simulated slant and total delays for another scenario in 2013, where a weather front arrived over Switzerland. We applied tomography and collocation, with the aim to assess their capability to detect this atmospheric structure. We selected two sub-networks from the GNSS network in Switzerland: 1) a NorthCH sub-network, where the stations are located at low altitudes and with a small variability in height and 2) the Valais sub-network (with the same configuration as in the 2016 scenario).

#### Real data in the Valais

**Fig. 1:**
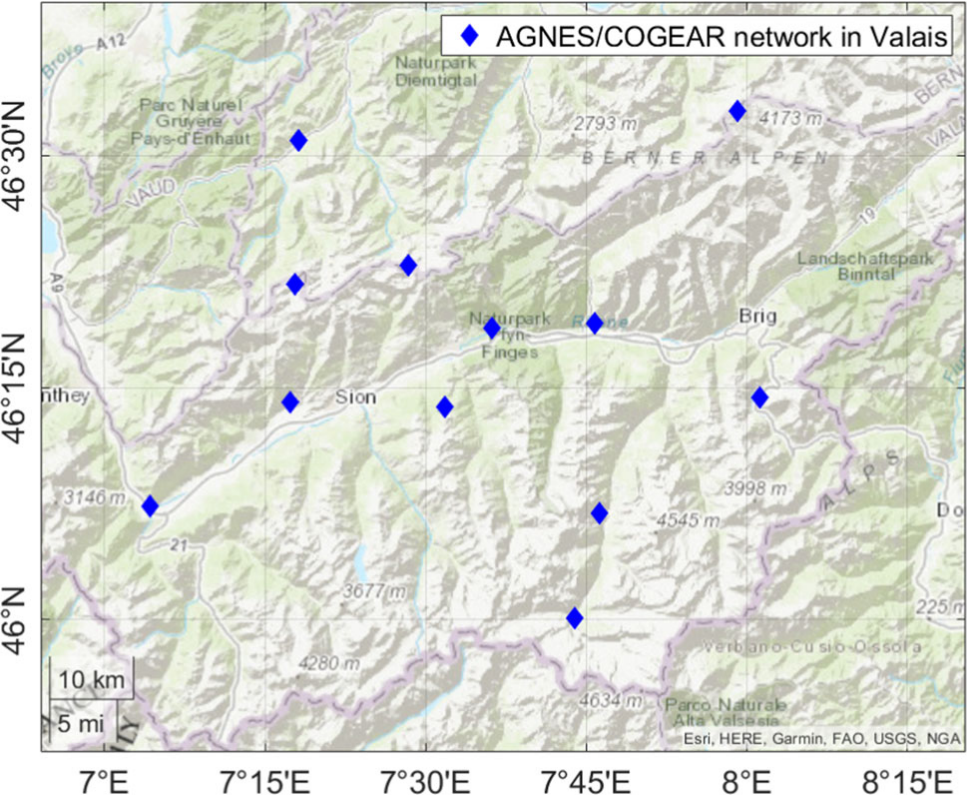
GNSS AGNES/COGEAR network in the Valais, Switzerland

The main area of investigation in this work is the Alpine region in the Valais, Switzerland. It is illustrated in Fig. 1, where the GNSS stations are marked in blue. This area is interesting, since there is a high variation of altitudes in the terrain and GNSS stations. This is a positive factor for tomography and collocation, since GNSS stations can sense the troposphere at different altitudes. However, the highly variable terrain results in highly variable refractivities. We utilized hourly zenith wet delays (ZWDs) of the AGNES/COGEAR network (Swisstopo 2019) for a 2-week period in August 2016, processed by swisstopo (Brockmann and Troller 2002; Brockmann et al. 2006).

#### Real data in the Upper Rhine Graben region

We also used zenith delays from the GURN (GNSS Upper Rhine Graben Network) in South Germany. This dataset is important to display collocation results and evaluate residuals in a very different scenario compared to the Valais use case. The GURN network consists of 66 stations in an area of about 5° × 5° latitude–longitude. The network is larger in size and has more stations. Furthermore, the area is rather flat, which has a different effect on the collocation results compared to the Valais scenario. The data were processed by the Geodetic Institute (GIK) of Karlsruhe Institute of Technology (KIT). For more details regarding this dataset, we refer the reader to Fersch et al. (2021).

### Preprocessing of refractivity fields to compare collocation and tomography

Tomography directly provides the refractivity at each voxel, whereas for collocation, we interpolate refractivities to the COSMO grid and then calculate the averaged refractivity of all points inside each tomographic voxel. This will be the collocation-based refractivity that we will compare with the tomography field. Similarly, we also compute the refractivity of the reference field from COSMO, which is used as ground truth. We have chosen a tomography grid of 24 horizontal and 14 vertical voxels. The a-priori field for tomography is based on a smoothed refractivity field which uses as input the NWM field.

Furthermore, we would like to point out that we computed the refractivity fields using ATom (Möller 2017), for two different slant inputs: 1) The slants are ray-traced along the COSMO grid (as explained in Sect. 2.5.1), and 2) the slants are computed from the ZTDs using a global mapping function (GMF) (Boehm et al. 2006). The latter case is more similar to slant delays estimated from GNSS processing, where mapping functions are utilized to map the estimated zenith delay into the slant direction. Since, in the first case, we are ray-tracing along the NWM grid, the slant delays may be much more accurate than those computed using a mapping function. This is the reason behind this comparison. From our tests, the comparison of the two retrieved fields reveals differences of few ppm for few voxels, for the 2 weeks in August, while most of the field is very similar. Therefore, in Sect. 3, we use only the ray-traced slant delays, for further comparisons and analysis.

## Results

This section presents the results for the individual test cases. Initially, we provide comparisons of refractivity fields retrieved through collocation and tomography. After analyzing 4D fields of refractivity from both methods, we investigate how they reconstruct the refractivity distribution within an approaching weather front. For selected scenarios, we present the results of a theoretical investigation, where we analyze the formal variances/covariances of the two methods. In the second part of our results, we focus on factors affecting the solution of least-squares collocation. After presenting the results from fine-tuning the stochastic models, we demonstrate the importance of station resolution and station heights in the collocation method.

### Refractivity fields retrieved from tomography and collocation

#### 2016 Valais use case

In this section, we present the refractivity fields obtained from collocation and tomographic processing.

**Fig. 2:**
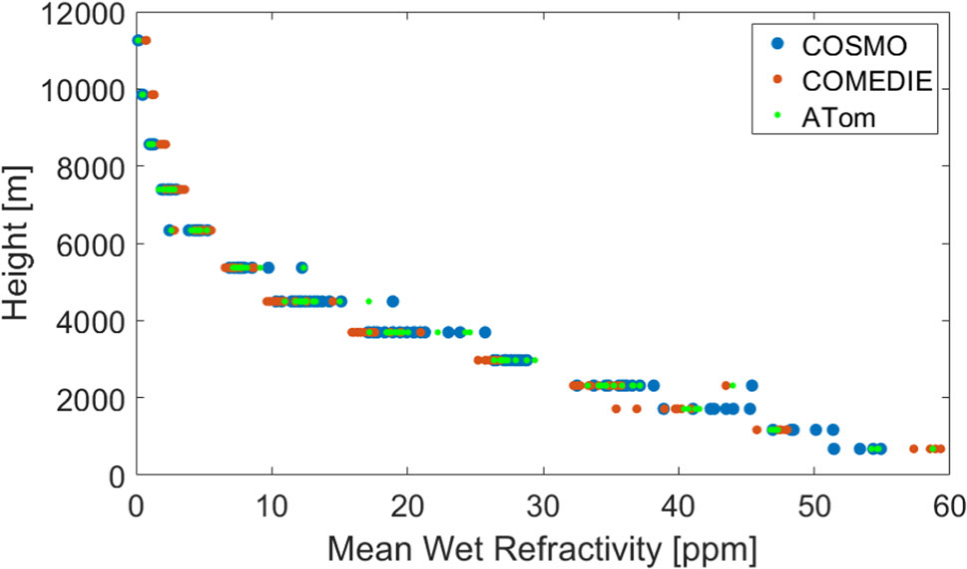
Averaged refractivities over 2 weeks in August 2016, plotted as a function of height for the ground truth COSMO refractivity, collocation solution (COMEDIE) and tomography solution (ATom)

In Fig. 2, we display the mean refractivity for each tomographic height, from the original COSMO field (in blue), COMEDIE (in red) and ATom (in green). We can notice that ATom produces refractivities with values closer to the original COSMO field, compared to COMEDIE. Indeed, a distinct bias is notable for COMEDIE, more obvious at high altitudes, whereas it is difficult to clearly see a bias for ATom.

**Fig. 3:**
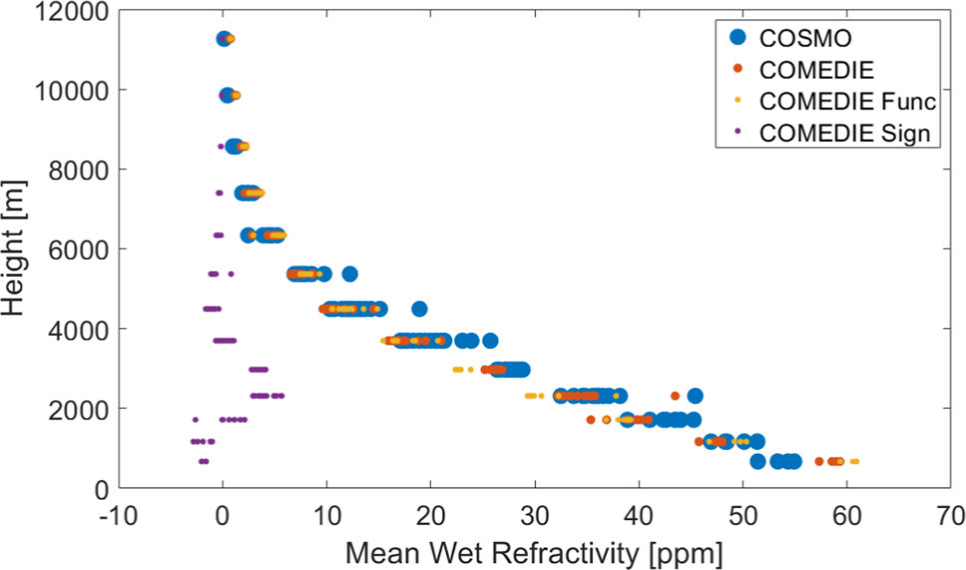
Averaged refractivities over 2 weeks in August 2016, plotted as a function of height for the ground truth COSMO-1 refractivity, collocation solution, collocation functional part and collocation signal part

In Fig. 3, we display the mean refractivity for each tomographic height, from the original COSMO field (in blue) and that from COMEDIE (in red). In yellow and purple, we show the functional and the signal part, respectively. In this figure, we want to display how the signal part 'helps' the functional part to get closer to the true COSMO value. For instance, at the lowest layer, the signal is negative, and the functional part is overestimated. Therefore, the total estimated value is closer to the COSMO value. On the other side, we can see how in the layers between 2000 and 4000 m, the functional part is underestimated, and the signal has positive values, bringing again the total value closer to the ground truth. At highest layers, above 7 km, we do not have any signal, since there are no stations at these altitudes (and the chosen signal correlation length in height is smaller than 2 km).

We calculated the differences of the refractivity fields retrieved by means of tomography and collocation, to the reference COSMO refractivity field. We point out that with collocation we can interpolate at any voxel; however, the differences are computed for both methods only for the voxels that tomography could resolve. We can conclude the following:

- For both methods, the difference to the reference refractivity is smaller at highest layers.
- For tomography, the largest differences to COSMO are experienced at the lowest layers up to ~ 4500 m.
- For collocation, very large differences to COSMO are notable at the lowest layers. This is explained by the fact that interpolation to the lowest layers is difficult for the mountainous terrain.
- For collocation, also very large values are notable at altitudes between 3500 and 5400 m. These are the altitudes, where we do not have any observations anymore.
- The differences between collocation and tomography (not shown here) can be more than 10 ppm for many epochs for some of the voxels below 6 km. From the average plots over 2 weeks, a few ppm differences (up to 5 ppm N-units) are calculated for the layers below 5 km, while the agreement is quite good in the higher layers.

**Fig. 4:**
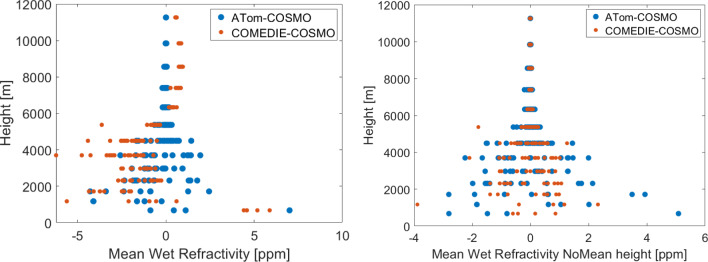
Tomography and collocation differences to COSMO refractivities, as a function of height, averaged over 2 weeks in 2016. Left: averaged total refractivity differences. Right: averaged refractivity differences after removal of the mean refractivity for each height

In Fig. 4, we plot the difference of the mean refractivity at different tomographic altitudes, for collocation and tomography, compared to COSMO. On the left, the total refractivity difference is shown. On the right, the difference after removing the mean for each height is displayed, i.e., for each of the three refractivity fields (ATom, COMEDIE and reference COSMO), we have computed the average of each tomographic height and subtracted it from the values of the refractivity at that height. While the COMEDIE solution is biased for different layers and performs worse (left subplot), the field where the mean refractivity for each height is removed (right subplot) has a smaller variability compared to the ATom solution. This is also confirmed in Table 1, where the statistics are displayed for the lowest nine layers. Therefore, the a-priori field, which has information regarding the mean refractivity for each layer, helps the tomography to have a better performance in terms of total refractivities. Collocation does not include this information, and it exhibits a bias compared to the reference. However, it can detect at a better level the variation of the residual refractivity among the voxels in one tomographic height, after removal of the mean refractivity for each tomographic height. In addition, another option would be to include an a-priori field in collocation as well; however, one of the main advantages of collocation is that it does not require any a-priori field, and this is why we opted not to include it.

**Table 1:** Statistics (in ppm, *N*-units) of refractivity field differences among collocation, tomography and the reference COSMO

Voxel height (m)		675	1170	1715	2314	2973	3698	4495	5372	6337
COMEDIE minus COSMO	Bias	5.0	− 1.7	− 3.0	− 1.3	− 1.5	− 2.3	− 1.9	− 0.6	0.3
	Std	8.2	6.9	3.6	3.3	3.0	3.8	3.2	2.2	1.4
	Std2	2.2	3.3	2.5	2.6	2.0	1.9	1.4	0.9	0.6
ATom minus COSMO	Bias	1.9	− 2.3	− 1.5	− 0.4	− 0.4	− 0.1	− 0.0	− 0.0	0.0
	Std	7.1	4.9	4.6	4.4	2.9	2.5	1.7	1.0	0.7
	Std2	6.2	3.9	4.1	4.2	2.8	2.4	1.6	0.9	0.7
COMEDIE minus ATom	Bias	3.1	0.5	− 1.5	− 1.0	− 1.1	− 2.2	− 1.9	− 0.6	0.3
	Std	9.2	5.2	3.7	4.0	2.8	3.5	2.9	2.0	1.3
	Std2	5.2	3.6	3.5	3.8	2.3	1.5	0.7	0.4	0.3

Std is the standard deviation of the differences of refractivities, while Std2 is the standard deviation of the differences after removal of the mean refractivity of the voxels in the same tomographic height. Voxel height is the ellipsoidal height of the center of the voxels

#### 2013 weather front scenario

**Fig. 5:**
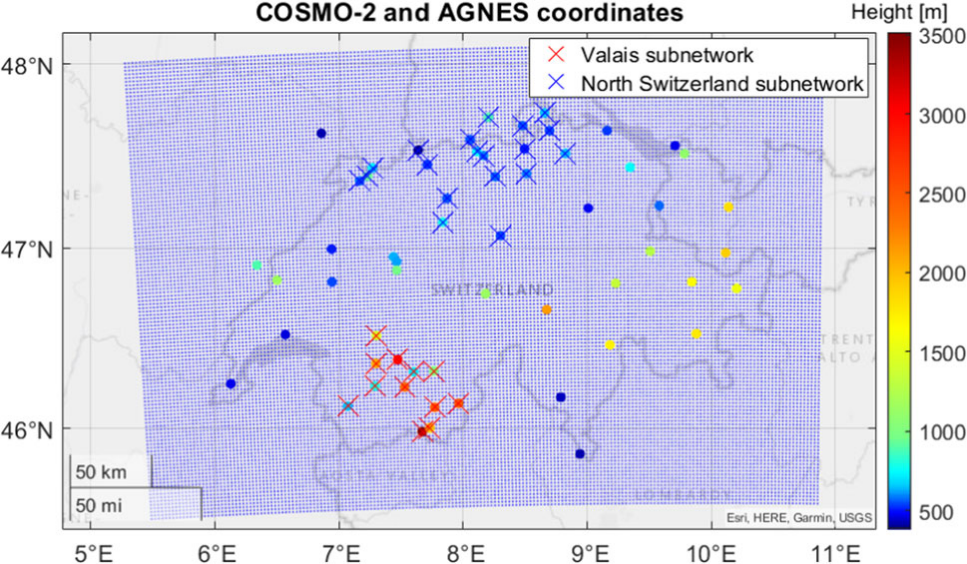
GNSS network in Switzerland (in dots) and two chosen sub-networks in the Valais and in the north of Switzerland (crossed stations). In blue, the NWP model grid, used to simulate the delays, is displayed

After a thorough comparison of tomography and collocation in the previous section, we now use both methods for a particular scenario in 2013, where a weather front arrived over Switzerland from the north. The NorthCH sub-network contains stations located at low altitudes with a small height variability, while the stations of the Valais sub-network have a large variability in height (with the same configuration as in the 2016 scenario). The chosen sub-networks are depicted in Fig. 5. The chosen tomographic grid is the same as for the 2016 scenario. We operated COMEDIE and ATom, for each sub-network separately.

**Fig. 6:**
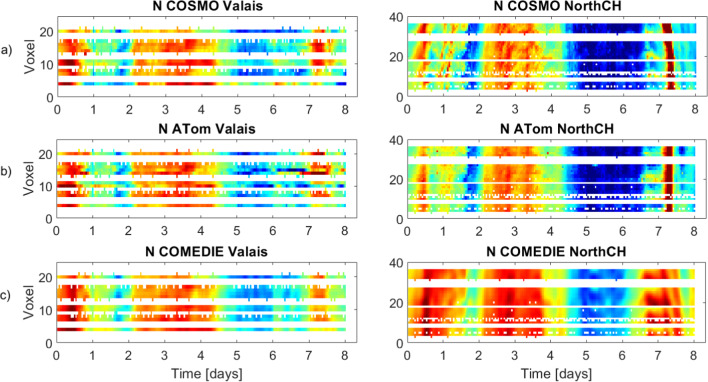
Time series of refractivity for the 24 voxels at ~ 2.5 km altitude: for the ground truth COSMO model (top subplots), estimated with tomography (middle subplots) and estimated with collocation (bottom subplots). On the left, the time series for the Valais sub-network are shown. On the right, the time series for the sub-network in the north of Switzerland are shown. The first epoch is May 27, 2013, at 00:00, and the last is June 3, 2013, 23:00. The refractivity values range from 15 N-unit (for dark red) to 35 N-unit (for dark blue)

For validation of the results, we show in Fig. 6, the refractivity for each voxel at ~ 2.5 km as a function of time for tomography, collocation and the reference solution, for both sub-networks. The weather front is over Switzerland at day ~ 4.5– ~ 6.5. From this figure, it is obvious that both methods can sense the mass arriving at both sub-networks. It also confirms the under- (over-) estimation of collocation for the NorthCH scenario, while collocation can better model the refractivity structure in the Valais. Note that our main objective is to prove the feasibility of both techniques to detect such atmospheric structures (at least at a general scale), and not to investigate the performance at every epoch and voxel. In this context, we point out that both techniques have the capability to detect atmospheric water vapor masses arriving over the GNSS network.

**Fig. 7:**
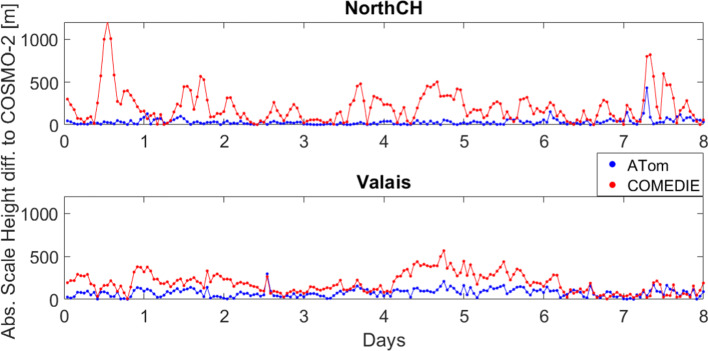
Absolute difference of the scale height obtained from COSMO refractivity profiles and estimated from ATom or COMEDIE. The scale heights for COSMO, ATom and COMEDIE are computed for profiles at the defined tomographic grid. The average scale height for each epoch is used to compute the differences shown here

Moreover, we investigated the scale heights for the two sub-networks. We computed the scale heights of refractivity profiles of COSMO, ATom and COMEDIE for profiles defined in the tomography grid. Figure 7 displays the difference between the scale heights computed from the COSMO profiles (considered as true values) and those estimated from collocation and tomography. A very clear agreement of the tomography scale heights (compared to collocation) to those of COSMO is visible. In case of collocation, the scale height differences for the Valais scenario are smaller than for NorthCH; this is related to the smaller ZWDs variations in the NorthCH scenario compared to the Valais. This confirms our assumption (mentioned in the previous sections of this paper) that the over- or under-estimation of refractivities, in networks with low variability in GNSS station altitudes, is affected by a worse estimation of the scale height.

### Formal variances/covariances analysis of tomography and collocation

**Fig. 8:**
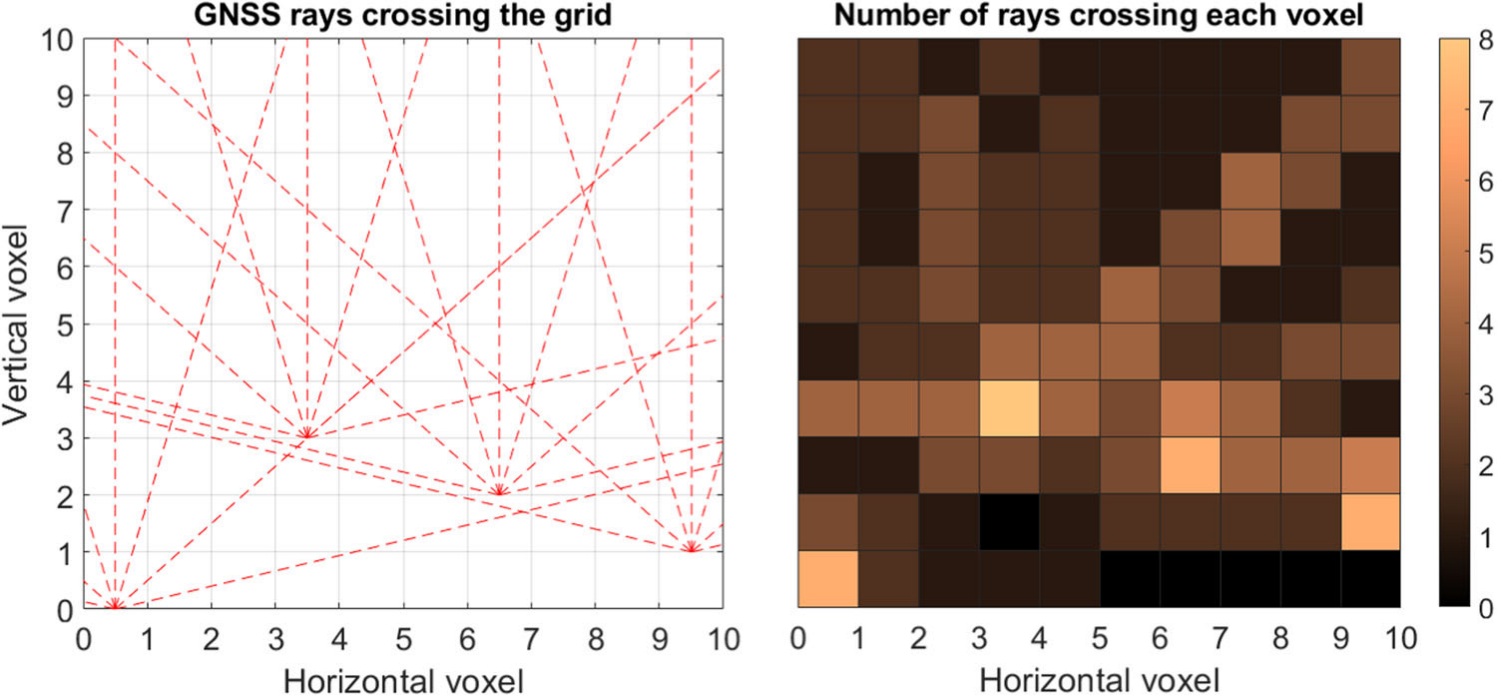
Left subplot: simulated GNSS network configuration with four stations and simulated rays at every 30 degrees of elevation, for a simplified 2D scenario. Right subplot: number of rays crossing each voxel

Initially, we display a configuration of four stations for which we have simulated rays at every 30 degrees of elevation. Figure 8 displays, on the left, the network configuration and the rays crossing the voxels, while, on the right, the number of rays passing through each voxel is visualized.

**Fig. 9:**
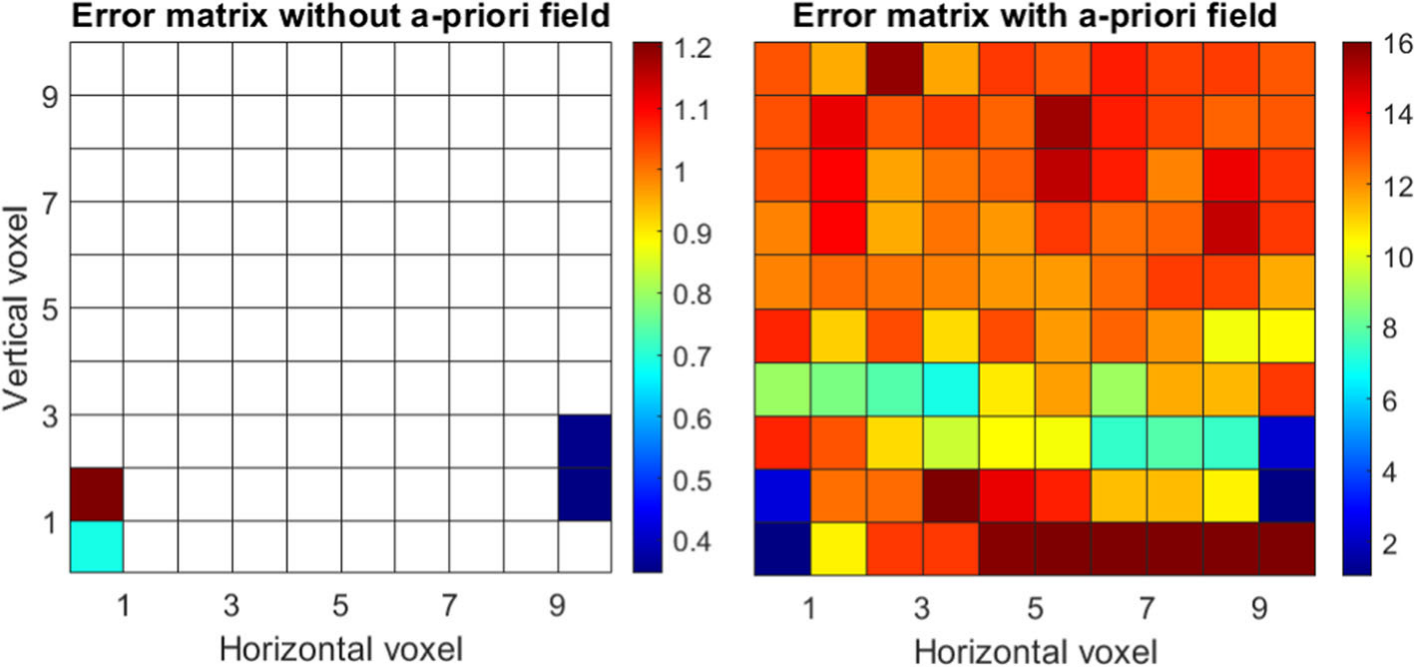
Tomography formal variances/covariances (ppm^2^) without assuming an a-priori field (left) and assuming an a-priori field (right). The large number of empty voxels results from the SVD (resolution matrix) analysis. Selecting a lower threshold for the SVD, more pixels in the ‘Error matrix without a-priori field’ are filled

We performed a single value decomposition to invert the A-matrix for tomography in case it was underdetermined. We computed the resolution matrix and defined (empirically) a value of 0.95 as a threshold for the resolution of the voxel refractivity. Figure 9 (left) visualizes the formal variances/covariances of tomography when we do not use an a-priori field and we can clearly see how most of the voxels cannot be determined with tomography without using an a-priori field. The right subplot of Fig. 9 shows the formal variances/covariances when we do use an a-priori field. In this case, all the voxels can be determined.

**Fig. 10:**
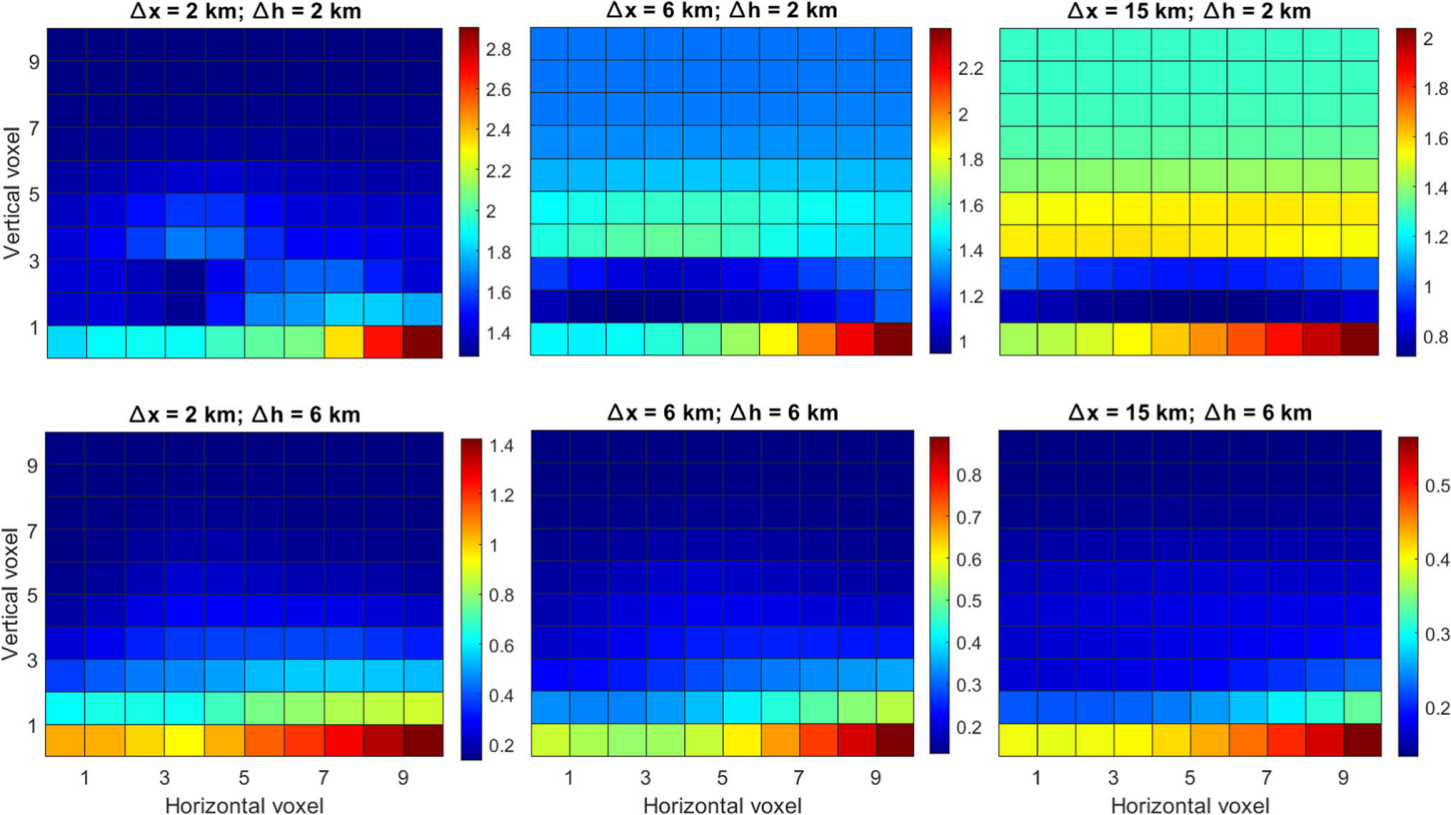
Collocation formal variances/covariances (ppm^2^) for different correlation lengths

Then, we computed the formal variances/covariances for collocation. For this case, we assumed different correlation lengths in the east and height components since the formal variances/covariances will directly depend on them. We can notice in Fig. 10 that the error matrix from the collocation approach is fully determined (for all cases), and it tends to have a dependency on the different height levels. The shape for all the different tunings tends to be similar, where smaller values are obtained at larger altitudes and close to the locations of the stations. Using large values for the correlation length in height (bottom subplots), clearly gives a height dependency to the solutions. When we use a small correlation length in height (top subplots), the formal variances/covariances become smaller in the vicinity of the stations. Using small values for the east component will also create more local gradients close to the GNSS stations (more visible in left and center top subplots). For all different tunings, there is clearly a different shape compared to the formal variances/covariances of tomography, which varies strongly even between neighboring voxels.

**Fig. 11:**
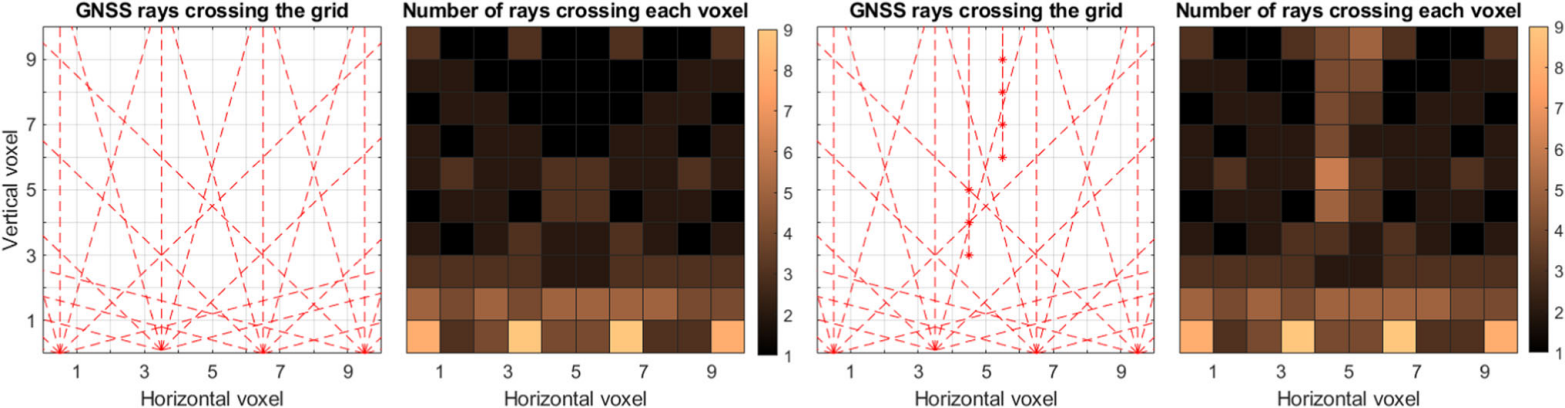
Two simulated GNSS network configurations, and the respective number of rays crossing each voxel. The first network has four stations in a flat area, and the slant delays are simulated at every 30 degrees of elevation. The second network has the same configuration as the first one; however, we have simulated 'profile' observations (in the vertical direction); the profiles contain only ZTDs (or refractivity), as if these were observed from radiosonde or radio occultation

In the following, we evaluated the formal variances/covariances of two more cases. In the first case, shown in Fig. 11 (on the left), we simulated a (nearly) flat network. In the second case, shown in Fig. 11 (on the center right), we also added profiles to this network; this could also be comparable to pointwise measurements from techniques such as radiosonde or radio occultation. Slant delays were simulated at every 30 degrees of elevation for the ground network for both cases, and for the profile only zenith delays are assumed. The number of rays crossing each voxel, for each of the simulated scenarios, is shown in the center left subplot (for the first case, without profiles of observations) and the right subplot (for the second case, including profiles of observations).

**Fig. 12:**
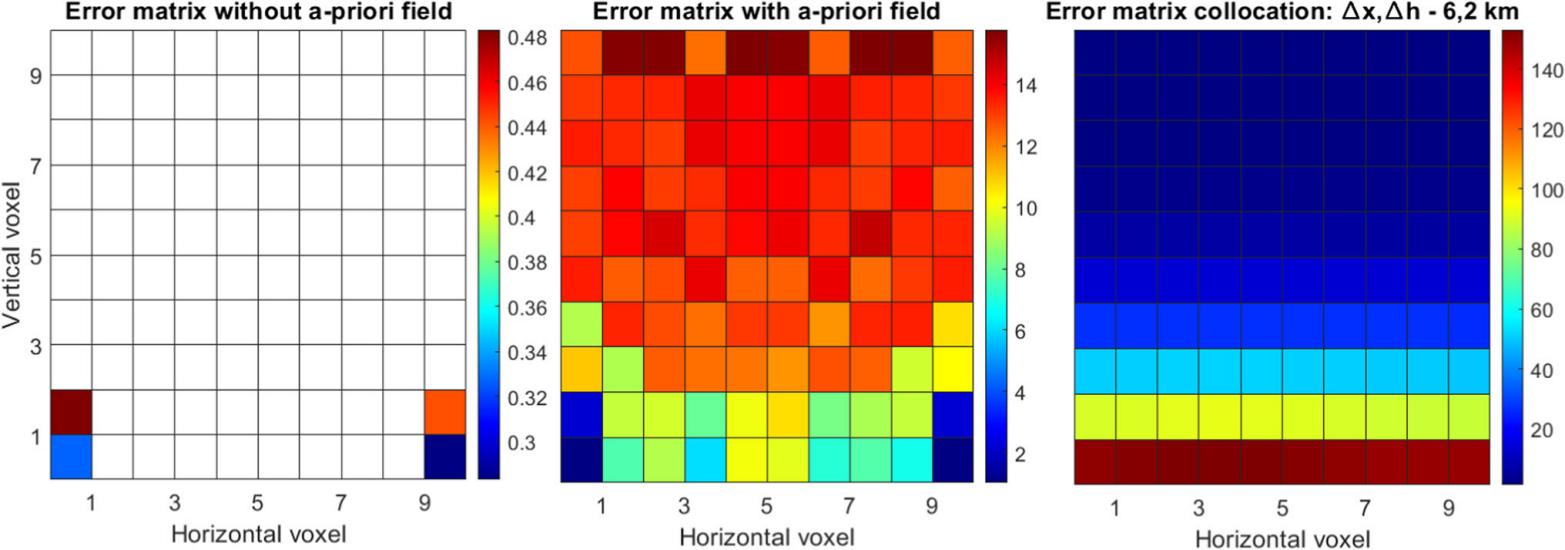
Formal variances/covariances (ppm^2^) for the network based on four stations at the bottom of the voxel model: from a tomography solution without assuming an a-priori field (left), tomography assuming an a-priori field (center), collocation (right)

Figure 12 displays the formal variances/covariances for the case where we use only the flat network, without additional profile observations. Only a few voxels can be determined by tomography without using an a-priori field (left subplot). We can clearly see the very different behavior of tomography (when we assume an a-priori field, center subplot) and collocation (right subplot). On the one side, the variation of the a-priori errors for collocation voxels is much smaller and height-dependent, slowly changing with altitude. On the other side, there is a large variation of the formal variances/covariances between tomography voxels, since they are differently crossed by the slant delays. The variation is quite large in both, horizontal and vertical coordinates.

**Fig. 13:**
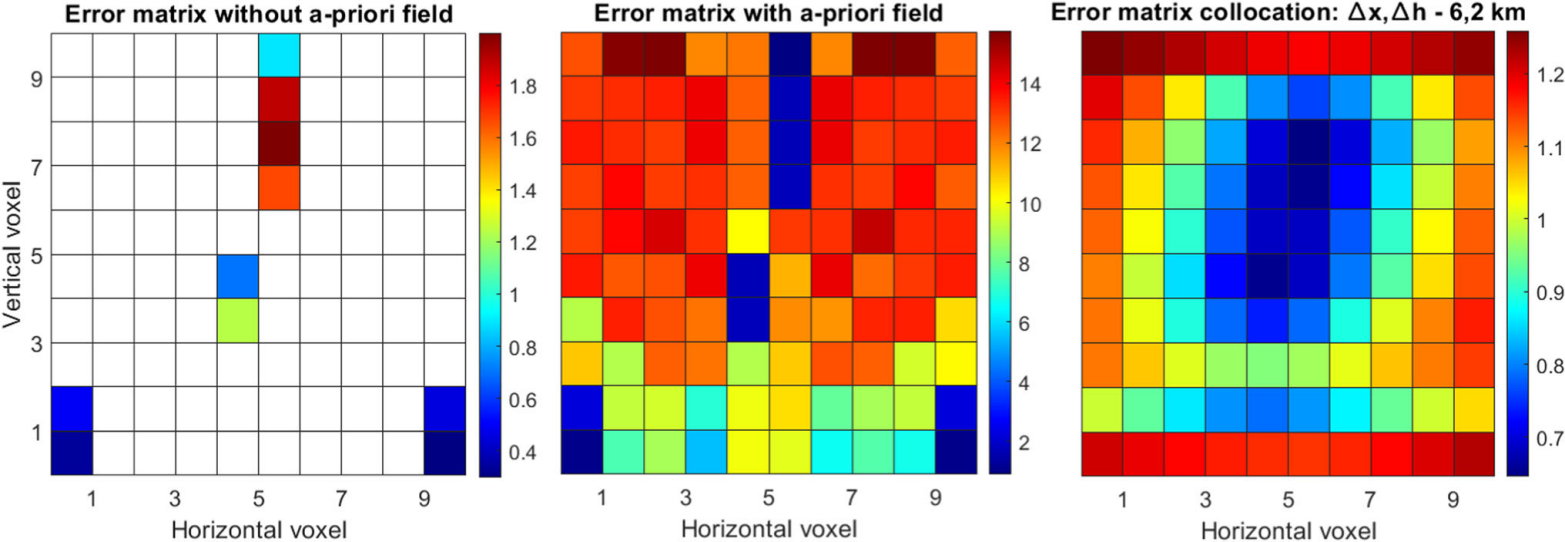
Formal variances/covariances (ppm^2^) for the flat network complemented by two vertical profiles: from a tomography solution without assuming an a-priori field (left), tomography assuming an a-priori field (center), collocation (right)

Figure 13 shows the results of the second case, where we accounted for the profiles of zenith delays as well. In the tomography solution without an a-priori field, the additional observations help to resolve those voxels where the zenith delays are located but have no clear effect on the other voxels. Including observations at higher voxels clearly changes the behavior of the formal variances/covariances of collocation (right subplot), as the errors become much smaller in general and especially at the locations of the profiles. The solution of tomography (when an a-priori background field is assumed) is clearly affected by the additional observations of zenith delays, which positively affects also the neighboring voxels. However, the overall error does not drop as dramatically as for collocation.

Our approach is based on conservative values for the resolution matrix. As discussed in Adavi et al. (2022), applying a more flexible approach to choose the threshold value can make the tomography results less reliant on the a-priori field. This means that more voxels can be resolved using tomography when we do not consider an a-priori field. For example, for the first case, shown here in Fig. 8, decreasing the threshold from 0.95 to 0.5 (or 0.35) increases the number of resolved voxels without considering an a-priori field from 4 to 9 (or 19). Considering a smaller threshold would lead to a singular error matrix.

We point out that we focus in this section on a high-level comparison of the formal variances/covariances. We mainly analyzed the shapes since the final values of the formal variances/covariances in tomography are highly dependent on the noise of the assumed a-priori field. In addition, in tomography, inter-voxel constraints can be added which could further change the shape of the error matrix. Finally, for this simplified case, we simulated delays at every 30 degrees of elevation, and we did not discard the slant delays entering the grid from the side. Our scope was to give a visual and simplified comparison of the two techniques, which emphasizes their general characteristics, such as height dependency of collocation and a-priori field inclusion in tomography. For actual 3D (or 4D) applications, where measurements are processed by means of tomography or collocation, these issues must be properly addressed.

From this section, we could draw a link to the results in Sect. 3.1.1, where the tomography solution, that considered an a-priori field, results in absolute values that are more similar to the reference refractivity field, while the accuracy of the collocation field changes with height. However, in the same layer, the error variation was smaller for collocation than for tomography. Similar behaviors could be seen from the a-priori error matrices in this section.

### Investigation of important factors in collocation results

This section focuses on the evaluation of several factors that affect the collocation performance. It aims to provide further insights into the assumptions and capabilities of collocation methods to retrieve refractivity fields based on ground-based GNSS tropospheric delays. Firstly, we provide an evaluation of the stochastic models assumed in collocation. Therefore, based on simulated datasets, we evaluate the importance of network resolution and height distribution on the retrieved refractivity field based on collocation. Finally, we analyze the collocation fields as well as the residuals of the observations, for two scenarios where two networks with very different height distributions, one with a good height distribution in the mountains and a network located in a flat area.

#### Results from the fine-tuning of the stochastic model

**Fig. 14:**
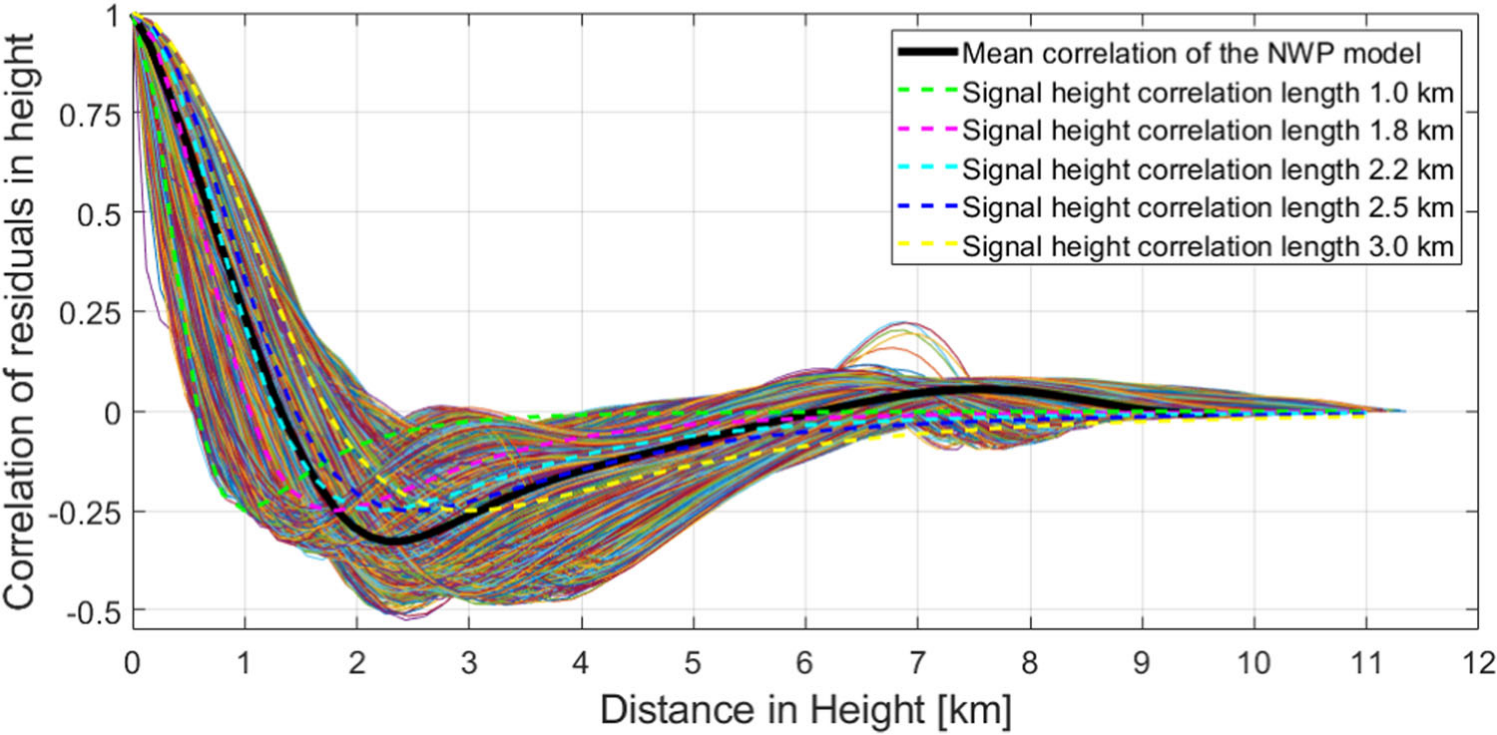
Correlations in height of NWP refractivities and of the signal model used in collocation, for different values assumed for the height correlation lengths

In Fig. 14, the correlations of the NWP model residuals are plotted in continuous lines for all ~ 9000 profiles available in the Valais region (see Sect. 2.5); the black curve is the mean over all curves. In the same figure, the correlations of our stochastic model for different assumptions of the correlation length in height are plotted in dashed lines. We can notice that for different locations within the NWP model, different stochastic models are suitable. Obviously, there is not a correlation length that would be perfectly suitable for all profiles (locations); however, we can approximate by finding the best fit to the mean NWP model correlation length, which is the black curve. A signal correlation length in height of ~ 2 km seems to be appropriate, which is the correlation length we usually use in our experiments for this area of investigation.

**Fig. 15:**
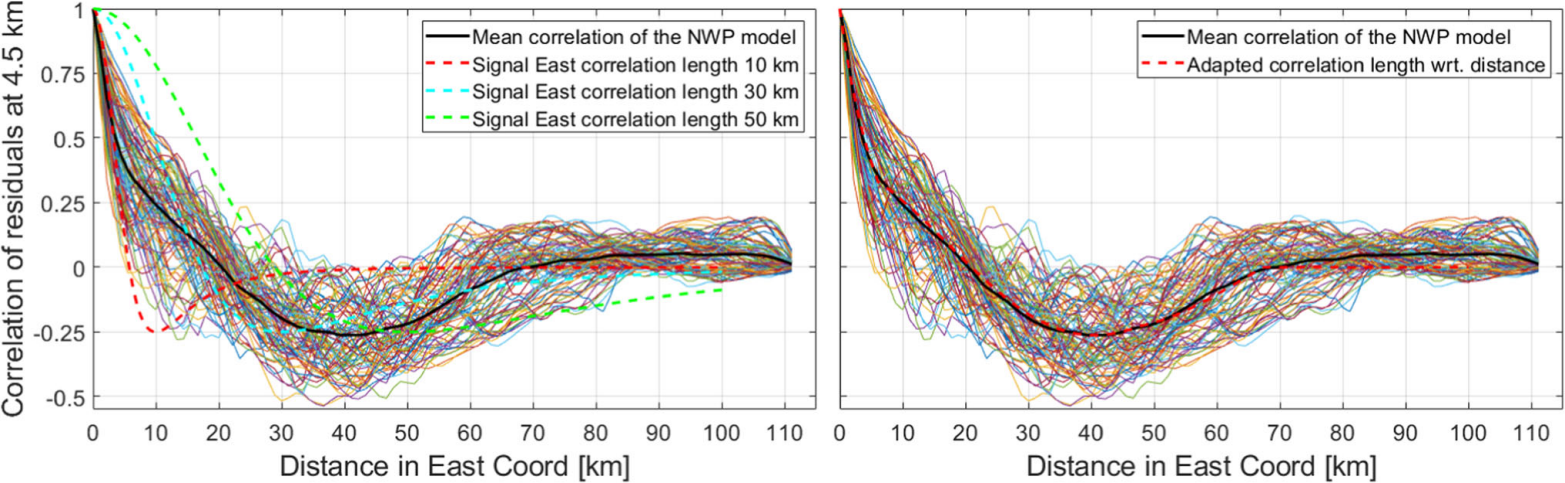
Left: spatial correlations of (residuals of) NWP refractivities in the east coordinate and correlation of the signal model for different correlation lengths in east direction. Right: spatial correlations of (residuals of) NWP refractivities in the east coordinate and correlation of the signal model for the east direction correlation lengths adapted to fit the mean NWP model correlation (using different correlation lengths for different distances)

In the left subplot of Fig. 15, we plot the spatial correlations of the NWP model residuals w.r.t. a linear function in continuous lines; in black, the mean of all curves is plotted. In dashed lines, we also plot the correlation of our stochastic model for different assumed correlation lengths in the east component. We can notice that a correlation length of 10 km fits best to the mean NWP-based correlations. This is the case because this curve (red curve) follows closer the average NWP correlation (i.e., the black curve), down to a value of 0.5. The value 0.5 is defined arbitrary, considering that the signal loses more than 50%. The other assumed correlation lengths (30 km and 50 km) fit better at other distances. However, since the values are below 0.5, we would lose most of the signal. We point out that it is important to capture the signals for the closest distances. This can be explained by the assumption of a rather dense network of GNSS stations and that the interpolation of the signal is more appropriate by giving higher weight to the ZTDs of the close-by station. Thus, the farther from the stations we are, the worse the signal can be modeled.

The right-hand subplot of Fig. 15 shows the case of adjusted correlation lengths for different distances, i.e., we assume different correlation lengths when we calculate the signal at different distances. We have done this experimentally by analyzing the mean correlation of the (residuals of the) NWP model. We note that this is done here only to illustrate that it is possible to modify the assumption of the signal model to capture a larger proportion of the correlated noise (e.g., when a-priori data are added).

It is important to point out that these simplified examples were displayed to show that our stochastic models have in general a reasonable agreement with the information from NWP models. Here, it is not our target to define the ultimate correlation lengths. However, through this data-based analytical evaluation, we aim to show the applicability and sensibility of our stochastic models.

#### Effect of network resolution and station heights

In this section, we utilize COSMO-simulated ZTDs at a very high resolution. We evaluate two components: 1) the impact of the network resolution and 2) the impact of station heights, on the retrieved refractivity field.

Table 2 shows that there is a degradation of the retrieved refractivity fields, especially in terms of standard deviation, when considering lower station resolution. The refractivity error increases by about 10%, from a resolution of 2 km to one of 10 km, below 3 km (from 4.7 to 5.3 ppm, see first line) and in the interval 3–6 km (from 2.9 to 3.2 ppm, see third line). At higher altitudes (6–11 km), the impact is not visible. The bias is large above 6 km, where for all cases, a value of about 5 ppm is computed. This is the case for collocation since it relies only on ground observations. Therefore, the computed scale height is only reliable up to the altitude of the highest station in the network.

**Table 2:** Closed-loop validation statistics for different network resolutions

Height/resolution (in km)		2	3	4	6	8	10
0–3 km	Std.dev [ppm]	4.7	4.7	4.8	5.0	5.2	5.3
	Bias [ppm]	1.5	1.6	1.6	1.5	1.6	1.6
3–6 km	Std.dev [ppm]	2.9	2.9	3.0	3.1	3.1	3.2
	Bias [ppm]	− 0.2	− 0.2	− 0.4	− 0.8	− 0.5	− 0.9
6–11 km	Std.dev [ppm]	2.1	2.1	2.1	2.2	2.1	2.2
	Bias [ppm]	5.0	5.0	5.0	4.9	5.0	4.9

We used NWP-based ZTDs (for 2 weeks in summer 2016) to simulate networks with different resolutions. The refractivity field was then computed by means of collocation and compared to the original refractivity in the NWP grid. The statistics from these differences are reported here

Table 3 displays that there is an obvious degradation in the retrieved refractivity field, from the scenario when all the points are used, to the scenario where only points at smaller altitudes are used. This is obvious for both, bias and standard deviation up to 6 km. However, we must point out the fact that, except when using only points at lower altitudes, the number of measurements is reduced as well, which further affects the results. The highest layers (6–11 km) do not seem to be affected.

**Table 3:** Closed-loop validation statistics for different maximal station heights of the GNSS network used in collocation

Height/station height (km)		< 1	< 1.5	< 2	< 2.5	< 3	All
0–3 km	Std.dev [ppm]	5.6	5.4	5.1	4.8	4.7	4.7
	Bias [ppm]	5.2	3.7	2.9	1.7	1.6	1.5
3–6 km	Std.dev [ppm]	3.7	3.7	3.6	3.4	3.2	2.9
	Bias [ppm]	0.8	0.5	0.5	~ 0	− 0.1	− 0.2
6–11 km	Std.dev [ppm]	2.0	2.0	2.1	2.1	2.1	2.1
	Bias [ppm]	4.9	5.0	5.0	5.0	5.0	5.0

We used NWP-based ZTDs (for 2 weeks in summer 2016) to simulate networks with different maximal heights. The refractivity field was computed by means of collocation, for each network, and compared to the original refractivity in the NWP grid. The statistics from these differences are reported here

#### Real data evaluations for Valais and URG

**Fig. 16:**
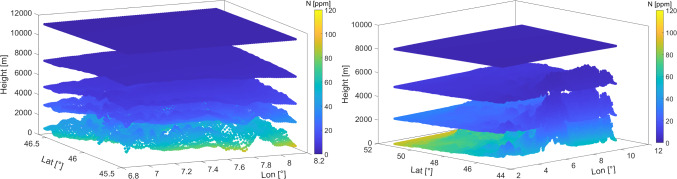
Wet refractivity fields retrieved by means of collocation for the Valais (left) and URG (right)

**Fig. 17:**
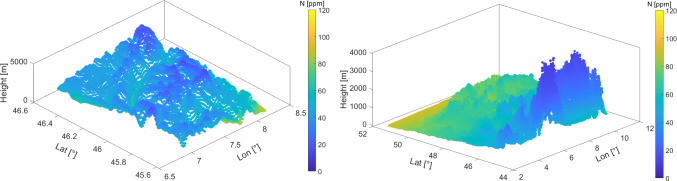
Wet refractivity field for the lowest layer retrieved by collocation for the Valais (left) and URG (right)

In this section, we present refractivity fields obtained from collocation, for two different scenarios, namely, for the Valais and URG regions. The collocation settings (assumed correlation lengths) have been adapted for each network. Figure 16 displays the refractivity at several layers, while Fig. 17 shows only the lowest layer field. For both scenarios, the refractivity field has lower values at higher altitudes. As explained from the stratification of the atmosphere, the refractivity field has a higher variability at lower layers. From Fig. 17, we can notice the dependency on the mountainous terrain in the Valais. For the URG scenario, the change of refractivity is relatively low, following the smooth topography changes in this area. The field reaches extreme values (lowest and highest, respectively) in the high-altitude area (in south–east) and in the lowest part of the valley (in north–west). We point out that these areas are outside of the GNSS network; therefore, the values are more prone to large errors. Indeed, collocation performs worse in case of extrapolation, compared to interpolation.

**Fig. 18:**
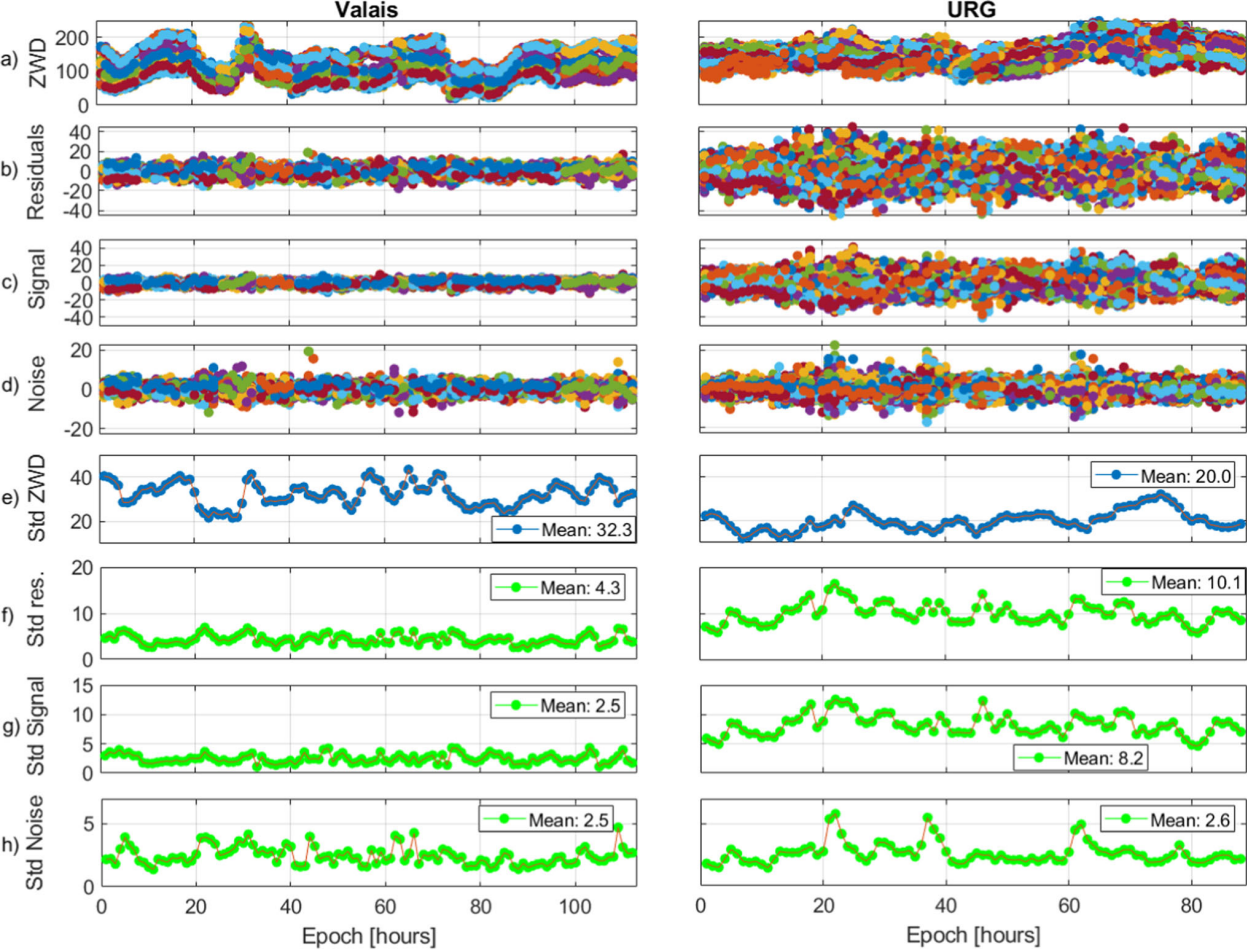
Time series for the Valais scenario (left) and the URG scenario (right). From top to bottom: **a** ZWDs, **b** ZWD residuals after collocation, **c** signal estimated with collocation, **d** noise estimated with collocation, **e** standard deviation of ZWDs, **f** standard deviation of ZWD residuals after collocation, **g** standard deviation of the signal estimated with collocation and **h** standard deviation of the noise estimated with collocation

To properly evaluate COMEDIE for these very different scenarios, we plot in Fig. 18 the ZWDs, the collocation residuals and the signal and noise, for each of them. In the lower subplots, we visualize the standard deviation of each component. We can notice the different ZWDs obtained for these two scenarios. In the Valais, they vary in an interval from 20 to 230 mm, with standard deviations in the interval from 21 to 43 mm. In URG, the ZWDs are in the interval from 72 to 252 mm, and their standard deviations are in the interval from 11 to 32 mm. The smaller standard deviations in URG are in line with the small altitude changes among the GNSS stations.

The very different delays, and altitudes of the GNSS stations, are, therefore, reflected in the collocation results, where we have smaller residuals for the Valais case. The two main reasons are: 1) We have a smaller area of investigation, therefore, the estimated functional parameters can better approximate the refractivity fields and 2) due to the very smooth topography in URG, the estimation of the scale height (see Eq. (8)) is less accurate. Indeed, having GNSS stations in very high altitudes leads to a better approximation of how the refractivity decreases with altitude. In the extreme case, where all stations are at the same altitude, it would be impossible to estimate this parameter, while the best-case scenario would be to have measurements distributed from the bottom to the top of the atmosphere. Therefore, the Valais scenario is closer to the best-case scenario.

A similar white noise of the observations (see Eq. (7)) of both areas leads to a larger amount of signal in URG. Here, the main scope is to illustrate the very different behavior of the functional part (modeling most of the ZWDs) for these two very different scenarios. If we are uncertain about the signal magnitude, one option is to increase the measurement noise by a factor of 2 or more; this allows to lower the signal-to-noise ratio and thus reduce the impact of the signal on the interpolated fields.

## Conclusions

In this paper, we presented the retrieval of refractivity fields from GNSS zenith and slant delays by means of collocation and tomography. For this purpose, we utilized the collocation software COMEDIE and the tomography software ATom.

In the current literature, the retrieval of refractivity fields by tomography (and several issues related to it) have been addressed thoroughly. More recent publications focus on enhancing the output of this technique via improving the assumptions, voxel constraints and lowering the impact of the a-priori field. On the other hand, the utilization of collocation for retrieval of refractivity fields has not yet been invested in such detail. Thus, the main objective of this work is to present collocation as an alternative solution and to encourage researchers in this domain to use both methods. Therefore, we focus most of the investigations of this study on collocation, and, in addition, we provide use-cases where we compare it to tomography.

Tomography is a well-established method, with which the refractivity field can fully be determined if enough rays cross each voxel. However, GNSS tomography must deal with the fact that many voxels may be underdetermined. Voxel parametrizations, a-priori field assumption and specific methods to deal with singularities are some of the approaches to obtain a reliable estimate from tomography. On the other side, collocation does not encounter these issues; it is based on parameter estimation from the measurements. Consequently, the accuracy of collocation depends on the models' capabilities to approximate the reality. Moreover, the parameters of the functional and stochastic models are chosen empirically; thus, its performance depends on their choice. In this study, the input for collocation is zenith delays, while tomography processes slant (and zenith) delays. Therefore, tomography uses more observations, which also contain more a-priori information regarding refractivity in the horizontal domain.

From the theoretical approach to evaluate collocation and tomography, we provide:

- An analytical analysis of the stochastic models of collocation. We simplified the models to one dimension, such as the vertical or one horizonal dimension, and we calculated the correlation of the signal with respect to distance. Then, we compared the correlation to the correlation of the NWP model, which is an independent source. We noticed that our model approximates well the NWP model behavior; however, we must be cautious to choose the right correlation lengths.
- An investigation about the formal variances/covariances of collocation and tomography. We provided the a-priori errors for a 2D case scenario, where we initially simplified the models accordingly. Then, we computed a-priori fields from collocation and tomography for different simulated scenarios. We can clearly notice how important it is for tomography to include the a-priori field, as otherwise many voxels cannot be resolved. The formal variances/covariances fields of collocation have a much smaller horizontal and vertical variability compared to tomography; indeed, much larger differences are visible for the formal variances/covariances of close voxels for tomography. Both methods benefit from relying on denser and better distributed stations.

We provided an experimental investigation of collocation. In our work, we used two GNSS datasets from two networks with a very different geometry. The first network is in the Alpine region in Switzerland, and the second one is in the URG valley. The first network has a large range of station heights, while the second one has a much smaller one. In addition, we used NWP data to simulate zenith and slant measurements and then considered the NWP refractivities as ground truth.

- Based on a closed-loop validation using numerical weather model data, we checked the impact of network resolution and network heights. As expected, the results verified the importance of having a very high-resolution network and stations well-distributed in height.
- For two GNSS scenarios, we provided refractivity fields and analyzed the residuals (and their signal components). We visualized how the refractivity fields follow the terrain at the lowest layer and decrease at the higher altitudes. Moreover, we noticed that in the URG scenario, the residuals are higher compared to the Valais case. We attribute this to the fact that the small heights (and height variability) of the stations in this scenario results in worse estimates of the scale height. Therefore, in this scenario, we aim to capture more structure from the signal. However, if we do not want to overestimate the signal, we can increase the standard deviation of the measurements by a factor k, and thus decrease the signal-to-noise ratio.

Based on simulated zenith and slant delays (from COSMO data), we performed a test campaign for 2 weeks in August 2016. We computed refractivity fields by means of tomography and collocation with a resolution of 3 h. We used the COSMO-based zenith and slant delays for the GNSS sub-network in the mountainous area in the Valais, Switzerland. Therefore, we compared both methods with the NWP fields which we considered as ground truth. We noticed that tomography performs better in terms of bias. However, when we remove the mean of each height level, both methods performed similarly (slightly better standard deviations for collocation). The a-priori field, which is not a necessary information for collocation, helps tomography to better estimate the bias. The overall agreement between the two methods is a few ppm at the lowest layers (in terms of average difference over 2 weeks), while above 5 km collocation and tomography agree very well.

We also evaluated collocation and tomography for a scenario in 2013, where a weather front arrived over Switzerland, for 2 days. The main objective was to understand if both methods can sense such atmospheric structures. To check the time series of both methods, we set up a scenario of 8 days, where we used two sub-networks. The first sub-network is located in the north of Switzerland, with small heights (and height variations), and the second one is again the sub-network in the mountainous area of the Valais. Once more, we utilized the NWP model COSMO to simulate slant and zenith delays. The general conclusion is that both methods can sense the water vapor mass, for both sub-networks. Tomography captures more structures in the north of Switzerland compared to collocation. Indeed, due to the low variation and altitude of GNSS stations in the north of Switzerland, the scale height parameter is estimated less accurately; using 'true' COSMO-computed scale heights, we also showed that the scale heights for NorthCH are less accurate compared to those of the Valais region. This leads to an over- or under-estimation of the refractivity by means of collocation. However, when the weather front arrives, looking at the time series of estimated refractivities, we notice that collocation estimates higher refractivity values (although less precisely), and thus senses the weather front. For the Valais scenario, where the station heights reach about 4 km, both methods capture similarly (and relatively well) the refractivity structures. Our main conclusion is that both methods (tomography and collocation) can be used to detect such atmospheric structures.

## Discussion

Previous works have addressed the effects of GNSS network resolution and height distribution on the solution of GNSS tomography. (Liu et al. 2019) use NWP observations to simulate different resolutions of the GNSS network. They show that increasing the resolution of the network from 1° to 0.5° improves the results by 11.2%. A resolution of 0.2° further improves the results by 15% compared to a 1° resolution network. (Troller 2004; Perler 2012) also highlight the importance of resolution and good height distribution of the GNSS stations as an important criterion to improve the quality of the tomography solution. The tomographic grid must be appropriately defined considering the height distribution of GNSS stations, and in addition, errors can be encountered in the solution if the grid is not fine enough to represent vertical changes of the water vapor. (Perler 2012) shows that: a) Higher network resolution can significantly decrease formal variances/covariances, b) the height distribution is a key property with a large impact on the formal accuracy of tomographic solutions, c) a network with well-distributed heights can decrease the formal variances/covariances limit and d) additional observations complementary to ground-based GNSS observations help to improve the vertical resolution in tomography.

Several studies have investigated the benefits of using collocation and tomography to combine GNSS ground-based delays with observations that have complementary information. One of the most interesting observations that complement ground-based GNSS are GNSS radio occultations, where refractivity profiles provide information regarding the vertical structure of the atmosphere. (Xia et al. 2013) use RO observations to further improve the solution of GNSS tomography, while in Hurter (2014) and (Shehaj 2023), simultaneous RO and ground-based GNSS observations are used to retrieve the refractivity field. Furthermore, to exploit the high horizontal resolution of InSAR observations, (Shehaj et al. 2020) use collocation to combine the tropospheric information from GNSS and InSAR. InSAR measurements hold relative information, i.e., the change of the slant delays with respect to a reference acquisition and a reference location in the image footprint. In other works, such as (Benevides et al. 2015) and (Zhang et al. 2021), spectrometer data are introduced to tomography to enhance the accuracy of the retrieved refractivity field.

This work aims at providing further insights into refractivity fields retrieved by least-squares collocation and their possible contributions to weather forecasting models. The assimilation of GNSS tomography products has been studied in the previous works. For example, (Hanna et al. 2019) assimilate tomography products in Weather Research and Forecast (WRF) model and validate the assimilated products with radiosonde observations. They show improvements in forecasting of relative humidity and temperature during events with heavy precipitation. Positive impacts of tomography in weather forecast are also displayed in Trzcina et al. (2020). Similar investigations are not available for refractivity fields retrieved by means of collocation. By studying strengths and weaknesses of collocation and comparing the collocation-based refractivity fields to tomography-based ones, we expect this work to give an indication of the value of collocation to provide 4D information of the tropospheric constituents to weather forecasts.

## Data Availability

The datasets generated and/or analyzed in this study are available from the corresponding author on reasonable request.
